# Supplementary material for: An Artificial Intelligence Dose Engine for Fast Carbon Ion Treatment Planning
Source: Int J Part Ther. 2026 Feb 19;19:101309. doi: 10.1016/j.ijpt.2026.101309 (PMC12985379; doi:10.1016/j.ijpt.2026.101309)
Supplement: Supplementary file 1 — Supplementary material [file mmc1.pdf]

Supplementary materials

Table S1 Weights used in the custom loss function

Parameter	w_{Mask}	w_{Depth}	w_{Region}
C-DoTA-d	0.4	1	0
C-DoTA- α	0	0	1
C-DoTA- β	0	0	0.4

Table S2 GPR 1%/1 mm results computed on the MC mask

Parameter	Value range	GPR (1%/1 mm) [%]				
		Median	Min	10%-tile	90%-tile	Max
D	0-2.5 Gy	99.76	92.06	98.70	99.96	100
α	0-1.6 Gy^{-1}	91.17	71.49	87.28	93.77	97.58
β	0-0.03 $Gy\text{-}^{2}$	91.37	71.69	87.66	93.80	97.69

Table S3 GPR 1%/1 mm results after 56 epochs with MSE. AI dose mask was applied to both GT and AI output.

Parameter	Value range	GPR (1%/1 mm) [%]				
		Median	Min	10%-tile	90%-tile	Max
D	0-2.5 Gy	99.61	88.66	98.02	99.94	100
α	0-1.6 Gy^{-1}	95.76	64.53	88.80	98.44	99.91
β	0-0.03 $Gy\text{-}^{2}$	95.39	62.65	87.90	98.20	99.61

Table S4 GPR 1%/1 mm results after 112 epochs with MSE. AI dose mask was applied to both GT and AI output.

**Parameter**	**Value range**	**GPR (1%/1 mm) [%]**				
		Median	Min	10%-tile	90%-tile	Max
D	0-2.5 Gy	99.66	91.54	98.46	99.94	100
α	0-1.6 Gy^{-1}	97.28	69.61	92.25	98.99	99.89
β	0-0.03 Gy^{-2}	96.26	65.93	90.61	98.56	99.73

Table S5 Additional evaluation metrics for voxel-wise comparison between FRED MC and C-DoTA outputs to show the impact of the two masks.

		**Median**	**Min**	**10%-tile**	**90%-tile**	**Max**
Voxels present in GT and not present in the prediction	Count	1479	292	983	2416	8530
	(in %)	8	1.32	5.48	11.42	23.38
	Total voxels in GT	19240	11531	14455	24566	38586
	Mean, Gy/10^7 primaries	0.02	0.01	0.02	0.02	0.03
	Mean± Standard deviation, %	1.17±0.17	1.08±0.07	1.14±0.12	1.25±0.29	1.83±1.31
Voxels present in the prediction and not present in GT	count	206	1	71	690	6973
	(in %)	1.15	0.00	0.37	4.23	21.09
	Total voxels in prediction	18032	10669	13577	22817	36523
	Mean, Gy/10^7 primaries	0.02	0.01	0.02	0.03	0.05
	Mean± Standard deviation, %	1.33±0.40	0.97±0.00	1.15±0.18	1.61±0.86	2.46±2.57

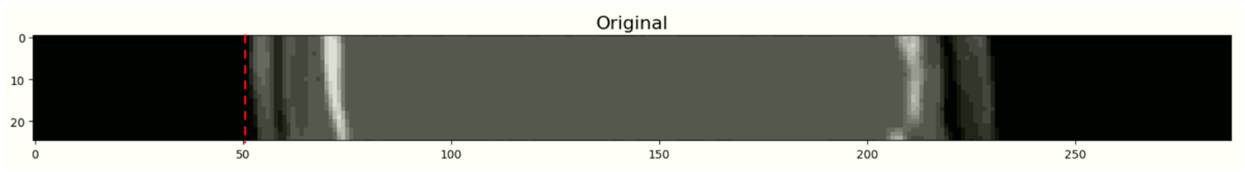

Figure S1 Example of a BEV-CT cut along the beam axis impinging from the left. The red dashed line indicates the cut of the proximal slices containing only air. X and Y axis of all images are in mm.

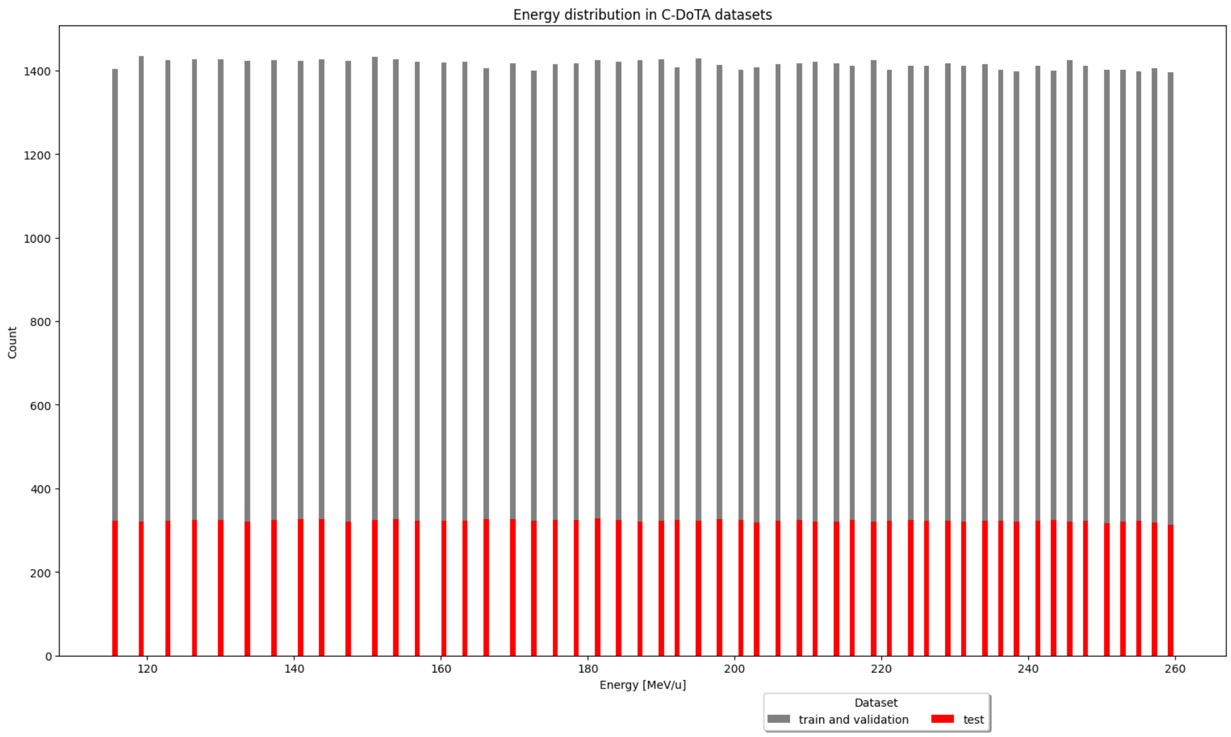

Figure S2: The number of samples in the C-DoTA database for discrete energies used at the CNAO facility, Italy.

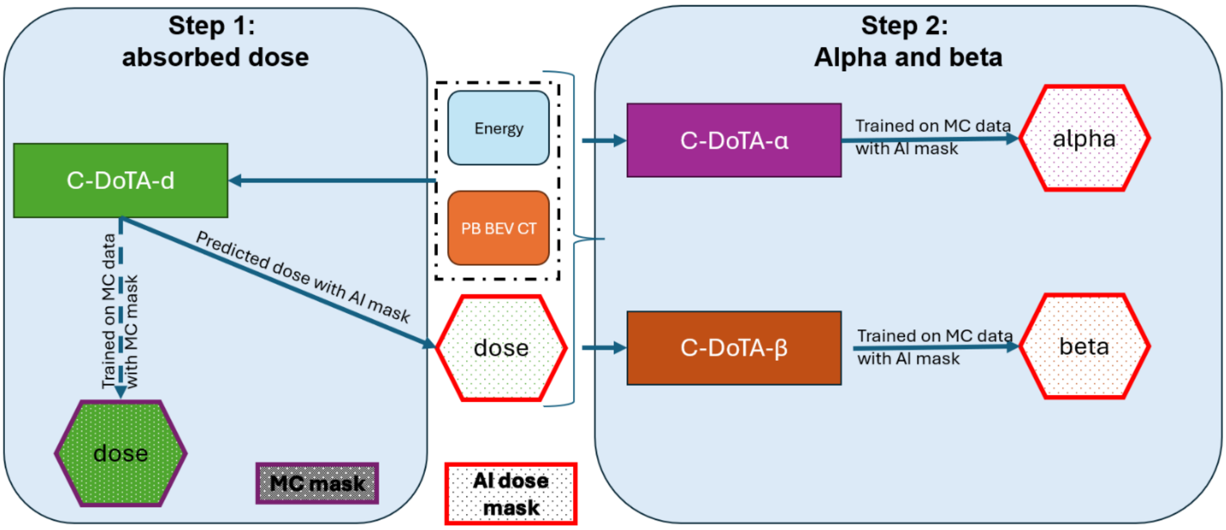

Figure S3 Data workflow. The output of the C-DoTA-d model is normalised to be used as an input.

The following Figures S4-7 and S9, S10 have the same structure: on the left, a longitudinal slice of the BEV CT is shown, followed by MC-GT and the C-DoTA prediction. The final column shows the difference between MC and C-DoTA. For better visibility, the maximum and minimum values of the color scale of the difference plots were set to the maximum between the absolute values of the 99.9%-tile and 0.1%-tile.

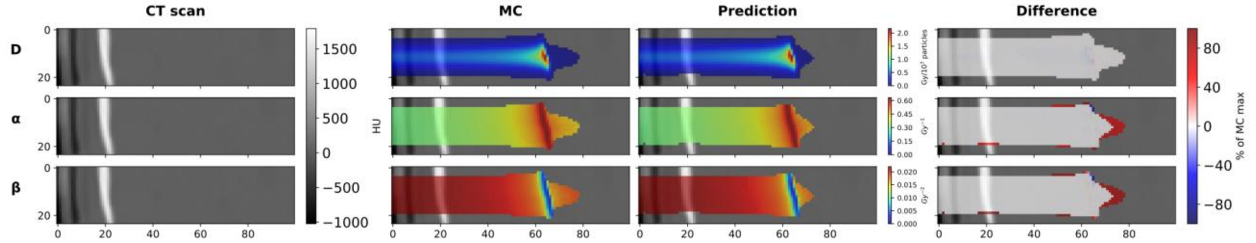

Figure S4 Best physical dose GPR result. To illustrate the difference between the applied masks, the MC results employ the MC mask, the C-DoTA results employ the AI mask. The difference shows all values without considering the masks. GPR 1%/1 mm: top row, dose:100%; middle row, alpha:100%; bottom row, beta: 99.87%. X and Y axis of all images are in mm.

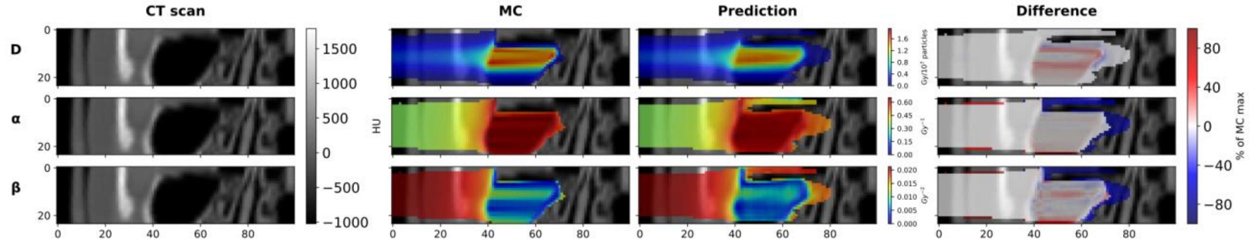

Figure S5 Worst physical dose GPR result. To illustrate the difference between the applied masks, the MC results employ the MC mask, the C-DoTA results employ the AI mask. The difference shows all values without considering the masks. GPR 1%/1 mm: top row, dose :92.06%; middle row, alpha: 89.16%; bottom row, beta: 90.62%. X and Y axis of all images are in mm.

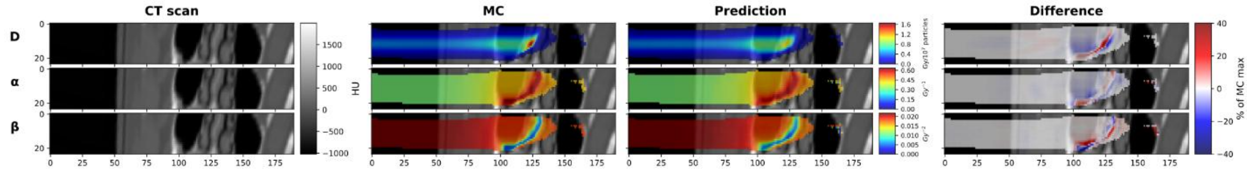

Figure S6 Worst result GPR for alpha. GPR 1%/1 mm: top row, dose: 95.28%; middle row, alpha: 86.36%; bottom row, beta: 87.76%.X and Y axis of all images are in mm.

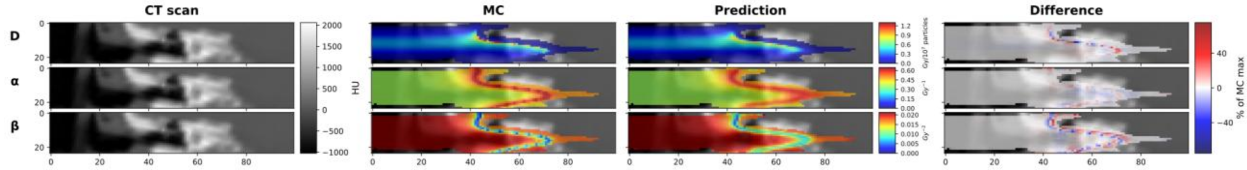

Figure S7 Worst result GPR for beta. GPR 1%/1 mm: top row, dose: 94.53%; middle row, alpha: 88.73%; bottom row, beta: 85.26%. X and Y axis of all images are in mm.

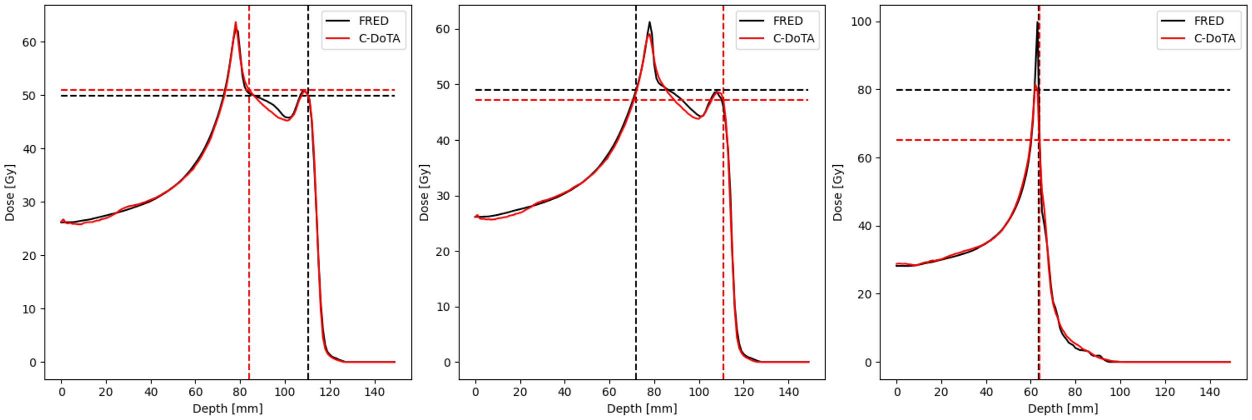

Figure S8 IDD Curve comparison. The left figure corresponds to ΔR80 max, the middle one ΔR80 min, and the right one to the maximum of ΔDmax.

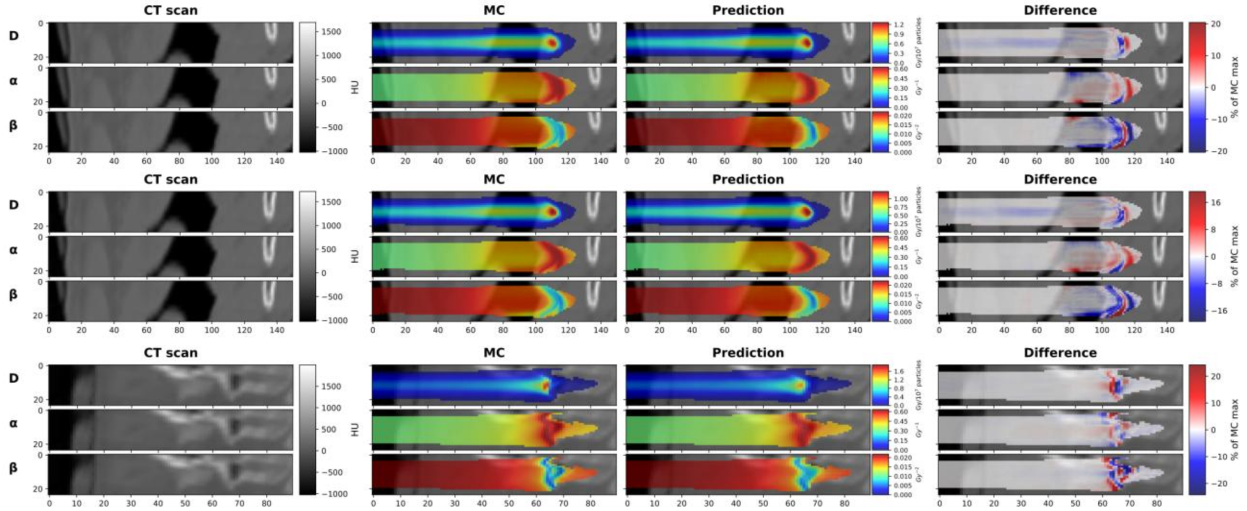

Figure S9 The top row corresponds to Figure S8 left panel, the middle row to the middle panel and the bottom row to the right panel. X and Y axis of all images are in mm.

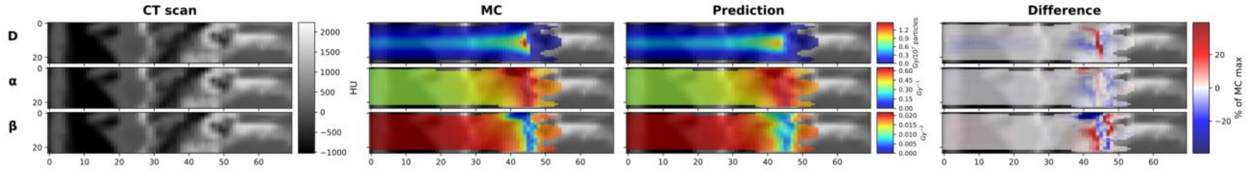

Figure S10 An additional test of the models on an inhomogeneous structure. GPR 1%/1 mm: top row, dose: 94.60%; middle row, alpha: 87.42%; bottom row, beta: 80.26%. X and Y axis of all images are in mm.
